# Endoscopic-assisted parieto-occipital interhemispheric precuneal transtentorial approach for microsurgical resection of vermian arteriovenous malformation: operative video and technical nuances

**DOI:** 10.3171/2020.10.FOCVID2067

**Published:** 2021-01-01

**Authors:** Kevin Zhao, Joseph Quillin, James K. Liu

**Affiliations:** Department of Neurological Surgery, Center for Skull Base and Pituitary Surgery, Rutgers University, New Jersey Medical School, Newark; Saint Barnabas Medical Center, RWJ Barnabas Health, Livingston, New Jersey

**Keywords:** arteriovenous malformation, microsurgery, endoscopic-assisted, parieto-occipital interhemispheric precuneal transtentorial approach

## Abstract

In this illustrative video, the authors demonstrate resection of a superior vermian arteriovenous malformation (AVM) using the endoscopic-assisted parieto-occipital interhemispheric precuneal transtentorial approach. Lateral positioning allows for gravity-assisted access to the interhemispheric fissure without retractors. The parieto-occipital trajectory is useful in patients who have a steep tentorial angle and avoids manipulation of the occipital lobe and visual cortex. In addition, the authors utilize an angled endoscope, which allows full inspection of the resection bed after AVM removal to visualize areas hidden from the microsurgical view to minimize the chance of residual disease in a deep corridor with multiple visual obstructions.

The video can be found here: https://youtu.be/hk9nIIdtqbI

## Transcript

This is Dr. James Liu, and I will be demonstrating the endoscopic-assisted parieto-occipital interhemispheric precuneal transtentorial approach for microsurgical resection of a vermian arteriovenous malformation.

**0:34 Patient History and Neurological Examination.** The patient is a 78-year-old female who presented with a sudden onset of headaches, nausea, and vomiting associated with truncal ataxia and loss of balance. Neurological exam was nonfocal without any neurological deficits.

**0:48 Preoperative Imaging.** CT scan showed a cerebellar hemorrhage in the superior vermis. CT angiogram demonstrated a possible superior vermian AVM draining into the vein of Galen. The AVM was fed primarily by the superior cerebellar arteries, confirmed on the 3D reconstructed images and diagnostic angiogram.

**1:10 Choice of Surgical Approach.** The surgical approaches to this region include the supracerebellar infratentorial approach. However, the steep slope of the tentorial angle makes this a difficult trajectory to access the AVM. Another approach to this region is the interhemispheric occipital transtentorial approach.^1–3^ However, this approach has an increased risk of visual field loss from retraction and manipulation of the visual cortex. Alternatively, we eventually chose the interhemispheric parieto-occipital precuneal transtentorial approach. This approach starts at a higher trajectory, which avoids occipital lobe retraction and also avoids the steep slope of the tentorium. It also provides a favorable direct trajectory to the AVM and the superior cerebellar artery feeders in the quadrigeminal cistern, as well as control of the draining vein going into the vein of Galen.^4–6^

**2:03 Lateral Position and Skin Incision.** The patient is placed in the lateral position with the right side down to allow gravity-assisted access to the interhemispheric fissure without fixed retraction. A linear skin incision is made over the parieto-occipital region based on the navigational plan. Image guidance is used to plan the optimal precuneal retrocallosal trajectory to the AVM, and a right parietal craniotomy is performed that crosses the midline.

**2:30 Dural Opening and Interhemispheric Exposure.** A C-shaped dural incision is made and reflected toward the superior sagittal sinus. CSF drainage with an external ventricular drain allows for brain relaxation and facilitates access to the interhemispheric fissure. After localizing the straight sinus with image guidance, a tentorial incision is made in a safe entry zone lateral to the straight sinus with a bipolar cautery and a no. 11 blade. This is done in an incremental fashion. A micro-Doppler is also used to confirm the position of the straight sinus. This ensures added safety to avoid sinus violation.

**3:07 Arachnoid Dissection.** The arachnoid membrane over the quadrigeminal cistern is opened using microscissors with careful spreading and sharp dissection.

**3:20 ICG Angiography.** Intraoperative ICG angiography is used to help identify the early draining vein going superiorly toward the vein of Galen.

**3:32 Corticectomy in Superior Vermis.** A corticectomy is made along the superior vermis using bipolar cautery. Careful bipolar dissection and gentle aspiration eventually exposes the nidus of the AVM.

**3:50 Extension of Tentorial Incision.** The tentorial incision is extended posteriorly to get better control of the posterior margin of the AVM.

**4:02 Evacuation of Hematoma Cavity.** We eventually reach the hematoma cavity. Evacuation of the hematoma gives added advantage of increased working room and control of the inferior margin of the AVM, as there are no arterial feeders in this region.^7^

**4:16 Circumferential Microdissection of AVM.** Dissection is now carried across the superior surface of the AVM toward the contralateral side. The nidus is carefully rolled toward the right side in order to visualize and dissect the brain-AVM interface on the left side. Care is taken to identify each arterial feeder, which are coagulated and divided sharply. Eventually, the draining vein is coagulated and divided toward the end of the circumferential dissection. As we inspect the opposite side, we visualize another distal SCA feeder, which is coagulated and divided to release the AVM from the resection cavity. It is important to inspect all surfaces of the AVM to ensure that all feeding vessels are detached from the nidus prior to final removal of the AVM.

**5:24 Endoscopic Inspection.** A 30-degree endoscope is used to inspect the resection bed. The vein of Galen is nicely visualized here. Angled endoscopy helps to look around corners that are not well seen with the microscope. There does not appear to be any residual AVM. The superior vermian vein is nicely preserved. We can now see the straight sinus at the base of the falx just medial to our transtentorial incision.

**5:59 Hemostasis and Closure.** Hemostasis is obtained and Surgicel is placed on the resection cavity. The external ventricular drain is then removed at the end of the case. Multilayered wound closure is performed in the standard fashion.

**6:15 Postoperative Imaging and Hospital Course.** Postoperative CTA and catheter angiography show no evidence of residual AVM. Postoperatively, the patient was neurologically intact and discharged to rehabilitation.

**6:28 Conclusion.** In summary, the interhemispheric parieto-occipital precuneal transtentorial approach with endoscopic assistance is a useful strategy to surgically treat superior vermian AVMs.

## Author Contributions

Primary surgeon: Liu. Assistant surgeon: Zhao, Quillin. Editing and drafting the video and abstract: all authors. Critically revising the work: all authors. Reviewed submitted version of the work: all authors. Approved the final version of the work on behalf of all authors: Liu. Supervision: Liu.
